# Prevention and treatment of intertrigo in large skin folds of adults: a systematic review

**DOI:** 10.1186/1472-6955-9-12

**Published:** 2010-07-13

**Authors:** Patriek Mistiaen, Meike van Halm-Walters

**Affiliations:** 1 NIVEL, Netherlands Institute for Health Services Research, PO Box 1568, 3500 BN Utrecht, The Netherlands; 2 LEVV, The Netherlands Centre of Excellence in Nursing, PO Box 3135, 3502 GC Utrecht, The Netherlands

## Abstract

**Background:**

Intertrigo in the large skin folds is a common problem. There is a plethora of treatments, but a lack of evidence about their efficacy. A nursing guideline on this matter had to be updated and broadened in scope to other health care professionals.

**Methods:**

A systematic review was performed. Thirteen databases were sensitively searched, supplemented by reference tracking and forward citation searches. All types of empirical research relating to the prevention or treatment of intertrigo were included. Study selection, assessment of bias, data-extraction and analysis were done by two independent review-authors.

**Results:**

Sixty-eight studies fulfilled the inclusion criteria. Only 4 studies were RCTs and even these had a considerable risk of bias. Study populations were generally small.

No studies were found about the prevention of intertrigo. The therapies concerned mostly the topical application of antimycotics, corticosteroids, antibiotics, antiseptics or a combination of these. Besides these pharmaceutical interventions, surgical breast reduction was also studied. Although most study-authors were positive, we could not draw firm conclusions about any of the pharmaceutical interventions. Even patients that received placebo intervention showed improvement. There is weak evidence that reduction mammaplasty may be helpful to treat inframammary intertrigo. All research found had considerable risk of bias, prohibiting firm conclusions.

**Conclusions:**

There is no evidence at all about the prevention of intertrigo and there is no firm evidence about its treatment. Well designed studies are needed.

## Background

Intertrigo is an inflammatory dermatosis involving body folds that develops through friction of skin to skin and is influenced by moist conditions. Intertrigo is found primarily in the inframammary, inguinal, abdominal and perianal skin folds. Besides these large skin folds, intertrigo may also affect minor skin folds. Intertrigo has been defined as 'an erythematous eruption in a skin fold, caused by warmth, moisture and chafing; it occurs most often in the skin folds under the breasts and in the groin' [1,2] or as 'a cutaneous inflammatory process on opposing skin surfaces' [3] or 'an inflammatory dermatosis involving body folds' [4].

The moist damaged skin can become secondary infected by bacteria or yeast and fungi. Visual symptoms of intertrigo are redness in mirror format and moist skin. Patients may complain of itching, burning feeling, pain and odor [1,2,5].

Prevalence of intertrigo in the large skin folds varies from 6% in hospital patients to 17% in nursing home clients and 20% in home care patients [6-12]. There are, to our knowledge, no figures about the prevalence of intertrigo in the general population, largely due to the fact that intertrigo has no specific ICD-10 or ICPC-score. Also there is overlap in medical taxonomies between intertrigo, dermatomycoses and bacterial skin infections.

A nursing guideline on the prevention and treatment of intertrigo was published in the Netherlands in 2004 [13]. For that guideline a systematic review [14] was performed containing references up to June 2002 and limited to interventions in the nursing domain. The review revealed that there was a plethora of interventions used but a dearth of well designed studies.

In 2009 it was decided that this guideline had to be updated and needed a broader scope, so it could be used by other health professionals as well. Therefore a new, updated and broadened systematic review was required. The aim of this manuscript is to give an overview of the evidence for preventing and treating intertrigo in the large skin folds of adult patients.

## Methods

A systematic review of the relevant international literature was performed. Two reviewers worked together during all steps of the review.

The search string 'Intertrigo [MESH] OR intertrig*' was used for Pubmed and adapted for the other databases. The following 13 databases were searched, with no limitations concerning date or language:

1. PUBMED (United States National Library of Medicine)

2. Embase (Excerpta Medica Database)

3. The Cochrane Library

(Cochrane Database of Systematic Reviews/Database of Abstracts of Reviews of Effects/Cochrane Central Register of Controlled Trials/Cochrane Methodology Register/Health Technology Assessment Database/NHS Economic Evaluation Database)

4. CINAHL (Cumulative Index to Nursing and Allied Health Literature)

5. SCI (Science Citation Index)

(Science Citation Index Expanded/Social Sciences Citation Index/Arts & Humanities Citation Index)

6. PICARTA (Dutch central library catalogue)

(NCC & OLC)

7. INVERT (Catalogue of Dutch nursing literature)

8. NHS-National Library for Health

9. LILACS (Latin American and Caribbean Health Sciences Literature)

10. SCIELO (Scientific Electronic Library Online of Brazilian scientific journals)

11. IMEMR (Index Medicus for the WHO Eastern Mediterranean Region)

12. AMED (Allied and Alternative MEDicine)

13. CAMbase (Complementary and Alternative Medicine)

In addition, references from relevant reviews or guidelines found during the above search actions were added to the initial database and a forward search was performed in SCI with the manuscripts that fulfilled the inclusion criteria. All searches were performed begin April 2009.

To be eligible for inclusion, the manuscripts had to meet the following inclusion criteria (in PICO(T)-format):

**P **atient: Adult patients with (a risk for) intertrigo, which may or may not be infected. The intertrigo has to be located in a major skin fold (inframammary, inguinal, perianal, abdominal or axillary) for the entire or part of the research population

**I **ntervention: all interventions aimed at the prevention or the treatment of intertrigo

**C **ontrol: no restrictions

**O **utcome: the absence/presence of intertrigo or increase/decrease in intertrigo symptoms, assessed by either the patient or a health professional

**T **ype research: all primary research performed in humans with a sample size >1

Exclusion criteria were research on children, ailments in minor skin folds (e.g. interdigital), diaper dermatitis, specific diseases other than intertrigo (*(bv. eczema, Hailey-Hailey disease, pemphigus, granuloma, psoriasis, acanthosis, Darier disease)*, in vitro research, research on animals, opinion articles, editorials and letters.

Inclusion criteria were assessed firstly on title and abstract (if available) and later on the full-text. The inclusion process was conducted by the two review-authors, independently of each other. Disagreements were discussed until consensus was reached.

Following data were extracted

- General (authors, journal, publication date, country, language)

- Research method (objective, design, analysis)

- Research population (age, gender, setting, sample size)

- Applied (control) interventions (type, dose, frequency, performer)

- Outcomes (type, instrument, time of measurement, frequency, assessor)

- Results & conclusions

The data were extracted by the first review-author and checked by the second.

Data were extracted only for those articles in which the entire research population consisted of patients with intertrigo in the large skin folds or, where these patients formed only part of the research population, for those articles that gave separate analyses for patients with intertrigo in large skin folds.

To assess the risk of bias, the included studies were first divided into comparative or non-comparative studies. Non-comparative studies were all those that used only a single treatment arm. The comparative studies were then further divided into a group where there was no (clear) randomization applied and a group of studies in which patients were allocated by randomization (RCTs). Without further assessment, all non-comparative studies and all comparative studies without randomization were classified as being at high risk of bias. Only the RCTs were further assessed on risk of bias with the screening score instrument that was introduced by NICE in 2009 [15]. This instrument screens for the risk on four types of bias ('selection bias', 'performance bias', 'attrition bias' and 'detection bias'). For each type three questions have to be answered and a conclusion formulated of either 'low risk of bias', 'unclear/unknown risk of bias' or 'high risk of bias'. Studies that scored 'low risk of bias' on all four components received a total score of 'low risk'; studies with 'high risk of bias' on all 4 components were scored totally as 'high risk' and all other combinations as 'unclear/unknown risk of bias'.

Risk of bias assessment was conducted by the two review-authors, independently of each other and disagreements were discussed until consensus was reached.

For the data-synthesis, studies were first categorized to prevention or treatment of intertrigo.

The treatments were than further categorized to (one or more) kind(s) of intervention: antimycotics, corticosteroids, antibiotics, antiseptics, combinations, placebo, surgical and other. For the comparative studies, a distinction was made between studies in which the intervention was compared to the same product but in another dose or frequency and studies in which an intervention was compared to another different intervention (either another therapeutic product, a placebo product or natural course).

It was planned to perform statistical meta-analysis with Review Manager 5.0 (risk ratios for dichotomous outcomes and weighted mean difference for continuous outcomes and both based on a random effects-model) on condition that there was enough statistical and clinical homogeneity.

## Results

### Seach Results and General Characteristics

The initial searches in the various databases yielded 1989 hits (Additional file 1). After eliminating duplicates 1,124 references were left for the inclusion process. All but 2 databases revealed unique hits.

Initial assessment of the references on title and abstract resulted in 316 references that were retained for full-text inclusion assessment. Of these, three could not be obtained in full-text. After full-text assessment, 136 references were excluded and 177 included.

Of these 177 references, 75 were manuscripts of empirical research on patients of whom all or some suffered from intertrigo in large skin folds. No studies were found with a population that received prevention for intertrigo. The other 102 references were either literature reviews or guidelines. The references of these were entered into the initial database and assessed on the inclusion criteria for this review. This resulted in 62 new references that were not part of the initial database. Twenty-two of these 62 references fulfilled the inclusion criteria and were added to the set for data-extraction.

Together with the 75 references from the initial search and the 22 from the reference tracking we had a set 97 relevant references. All these were than entered into the Science Citation Index for a forward search to find manuscripts that referred to one of these 97 references. This resulted in 58 new references that were assessed on the inclusion criteria, which meant an additional six could be added to the set for data-extraction (totaling 103 references).

These 103 references referred to 100 different studies. Only in 68 studies were separate analyses for intertrigo in large skin folds presented; in the remainder the intertrigo in large skin folds was studied together with other diseases (e.g. all kind of dermatomycoses) or with other locations (large skin folds and interdigital ailments taken together), meaning that no meaningful data extraction or analyses could be done for this systematic review.

**Figure 1:**
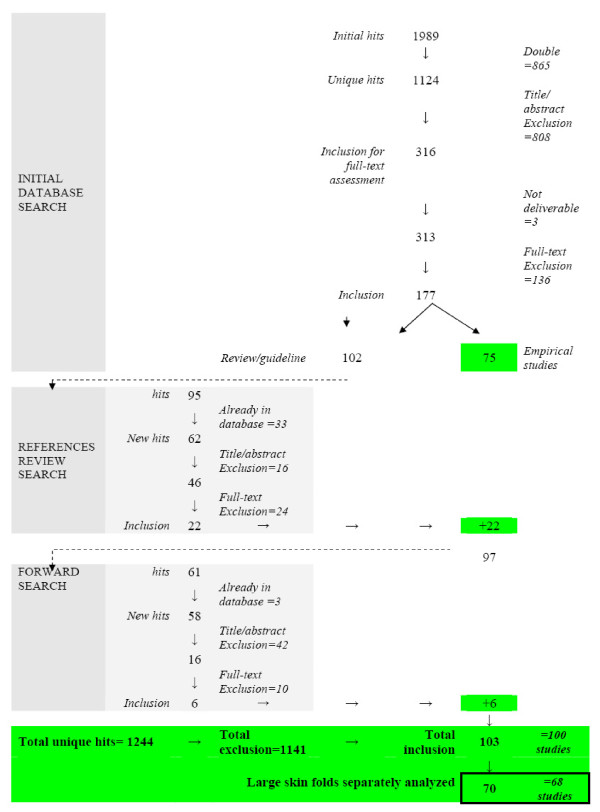
**Flow chart inclusion process**.

Accordingly, the final set for data extraction and analyses consisted of 70 references, representing 68 studies (the two manuscripts of Chapman [16,17] and the two of Spector [18,19] are respectively discussed as a single study). The flow of the inclusion process is depicted in Figure 1.

The 68 included studies date from 1954 up to 2008. Half of them date from before 1985 and 10% have been published in the last 5 years. The studies originate from 27 different countries; most are from the USA (16 studies), France (9 studies) or Germany and the UK (both 6 studies)). The studies were published in 51 different journals en in 9 different languages (for these the review-authors sought assistance from colleagues who master those specific languages).

### Research Designs and Risk of Bias

The 68 studies concerned 52 non-comparative and 16 comparative studies. Most of the non-comparative studies used a post-only design. Of the 16 comparative studies only four used a randomization procedure to allocate the patients to the research conditions. These four RCTs [20-23] were assessed on the risk of bias with the NICE-instrument [24]. None of the four complied with the criteria to qualify as 'low-risk on bias'. The largest problem in these four studies concerned the absence of valid and reliable instruments and methods by which outcomes were measured, resulting in high risk of detection bias. All four RCTs were judged as 'unclear/unknown risk of bias'. All other studies than the RCTs were judged as 'large risk on bias'.

In six of the 16 comparative studies a placebo intervention was used in one of the comparison groups. No study used natural course for comparison.

The number of patients with intertrigo in the large skin folds was rather small (median = 21; mean = 52.7; sd = 78.9; min-max = 1-406). Due to these small samples, there is a priori a considerable chance that the studies could not detect significant differences between groups.

The scientific quality of the studies was hampered even more by the fact that almost all the studies measured their outcomes with self developed instruments that had unclear psychometric properties.

In addition, outcomes were measured at many different moments after the start of therapy, making comparisons between studies difficult.

Another methodological problem was that most papers did not, or not clearly, present the extent to which the interventions and control conditions were implemented as was intended, i.e. in regard to dose, frequency or actor. For example, 'ointment 1 to 4 times a day' or 'for a period of 1 to 4 weeks' are not clear descriptions of an intervention. Likewise, potential confounding co-interventions, e.g. hygienic care of skin folds, were generally not described.

The applied statistics in the papers were not always scientifically sound and accurate. For example, frequencies were sometimes presented for different groups but not statistically tested for significant differences; or sometimes patients that did not respond to their allocated treatment were switched to an alternative group or dropped from further analysis, and in the multicentre studies no multi-level analyses were applied.

Only a minority of the studies declared that there was an approval from an ethics review committee or that patients were asked for informed consent. The financing source was mentioned in only 7 of the 68 studies; in all cases this involved a pharmaceutical company.

All things considered, this means that almost all of the 68 included studies lacked scientific rigor and all the results have to be considered with great caution.

### Applied Interventions

No study addressed the prevention of intertrigo. All studies concerned the treatment of existing intertrigo and mainly the treatment of intertrigo lesions that were already infected (n = 40). In only three studies was it explicitly stated that the research concerned intertrigo that was not yet infected [16,25,26]. In the remaining studies the phase/severity of the intertrigo was not clear.

A large variety of therapeutic interventions was applied, with the majority consisting of some kind of topical pharmaceutical treatment (antimycotics, antibiotics, antiseptics, corticosteroids or a combination of these); and in some studies a surgical intervention was employed (Additional file 2).

All therapeutic interventions concerned topical application except for three studies [23,27,28] in which antimycotics were given orally. Within the main categories many different products were used and also in various doses, varying frequencies and for varying lengths of time.

### Outcome Measures

The outcome measures applied in the studies can be roughly categorized into (degree of) healing, (decrease in) symptoms (number and/or severity), and tolerance and adverse effects of products

These outcome measures were operationalized in many ways, measured by various (mostly self-developed) instruments and assessed for instance in some studies by laboratory testing, or by physicians, and in other by patients, and measured at different moments. The (degree of) healing was the most frequently used primary outcome.

### Data Synthesis

The large variety of interventions, even within a specific category, and the large variety in outcome measures and measurements caused to large heterogeneity to attempt statistically pooling of results. Therefore results are presented below and in the Additional files 3, 4 and 5 only in a merely descriptive fashion.

#### Antimycotics

Antimycotics were used in 38 studies [20-23,27-60]. In 25 studies the antimycotic was used as a sole intervention; in the other cases the antimycotic was part of a combination. Only the studies with the antimycotic as a sole intervention are discussed here. Ten studies were comparative, either with the same product but in different form or using another way of administration (n = 4), or with another therapy (n = 4), or with a placebo-intervention (n = 5). Randomization was used in three of these 10 comparative studies.

The mean sample size of patients with intertrigo in the large skin folds in the 25 antimycotic studies was 34.7 patients (sd 59.2, min-max 1-245, median 18).

The applied antimycotics were categorized according to the Dutch Pharmacotherapeutic Compass: antimycotic antibiotics (amphotericine-B [36], nystatine [39,40], candidicin [37], pimaricin [30]); imidazoles (ketoconazole [27,28,31], micozanole [57], bifonazole [58], clotrimazole [20,22], econazole [20,22,43,49,51,52], thiabendazole [56], tioconazole [53,55]); triazoles (fluconazole [23]); allylamine (naftifin [45]) and other antimycotic (cyclopyroxolamine [46,54], fluorocytosin [39], dibenzthieen [38], buclosamide [41]).

With the exception of three studies [23,27,28] in which the antimycotic was given orally, all others concerned topical application.

Additional file 3 shows the effects per kind of antimycotic and per kind of study design. For the purpose of overview, doses and frequency are not shown; for each study the number of patients with intertrigo in the large skin folds is presented between brackets (the total population studied may have been larger, if it included also people with intertrigo in other locations or people with other ailments).

Taken into account the weak study designs, no strong conclusions can be drawn. It is remarkable however that the study-authors all drew positive conclusions about the products they studied. So one could say that there is tentative evidence that antimycotics do work in case of infected intertrigo and they are better than placebo interventions. There is no basis to state that one specific preparation is better than another, and there is no reason to assert that oral antimycotics work better than topical ones or vice versa.

#### Corticosteroids

In 17 studies [21,25,29,30,32,34,35,42,44,47,48,61-66] (Additional file 4) a topical corticosteroïd was used, in four of these as a sole intervention [21,25,61,64]. Only one study [21] applied randomization to allocate patients.

The mean sample size of patients with intertrigo in the large skin folds in this group of studies was 27.7 patients (sd 33.9, min-max 7-78, median 13).

The four studies in which corticosteroids were used as a sole intervention suggest that corticosteroids do have some effect, and the Hedley study [21] suggests that hydrocortisone is as effective as the combination of hydrocortisone + miconazole. However, since there are no placebo controlled studies, only one comparative study, and the study populations are small, no firm conclusions can be drawn about the usefulness of corticosteroid application to intertrigo lesions.

#### Antibiotics

An antibiotic was applied in eight studies [30,32,33,35,48,50,62,65]. However, in all cases the antibiotic was part of a combined preparation (e.g. combined with a corticosteroid). So no conclusions can be drawn about the effectiveness of antibiotics on their own.

#### Antiseptics

In five studies [29,59,63,66,67] a topical antiseptic was used, but in only one [67] as a separate intervention. Bonnefoy et al. [67] compared eosine to the application of cicalfate in symmetrical lesions of the large skin folds in 49 patients; the allocation was not randomized. The general effectiveness was judged to be very good in 40/49 lesions treated with cicalfate versus 31/49 treated with eosine.

Therefore, there is insufficient evidence to draw conclusions about the effectiveness of antiseptics.

#### Combined preparations

In 18 studies [21,29,30,32-35,42,44,47,48,50,59,60,62,63,65,66] (Additional file 5) a topical combined preparation was used. Four studies were comparative: two [59,66] compared a combination to another combination, one [30] to an antimycotic and one other [21] to a corticosteroïd.

The combinations usually consisted of an antimycotic and/or a corticosteroid and/or an antibiotic and/or an antiseptic.

The mean sample size of patients with intertrigo in the large skin folds in this group was 27.2 patients (sd 32.8, min-max 2-124, median 15).

Although the study-authors claimed that the combinations worked, no firm conclusions about any of the combinations can be made due to weak research designs and the small sample sizes.

#### Placebo interventions

Six studies [20,22,36-38,68] compared an intervention to a placebo intervention, two of the studies [20,22] were RCTs. The mean sample size of patients with intertrigo in the large skin folds in this group was 57.3 patients (sd 92.6, min-max 4-245, median 21.5).

The placebo intervention was the same as that used as the basis component for the therapeutic comparison, except for the of McMahon study[68] in which water and soap were used as placebo intervention. The placebos were compared to econazole [20,22], clotrimazole [20,22], amphotericine-B [36], candidicin [37], dibenzthieen [38] or to water and soap with talcum powder or with gauzes or with barrier cream or with hydrocolloïd [68].

The Cullen study [20] contained six patients in the placebo group, one of whom showed completed and three others partial healing. In the Miura study [22] of 245 lesions, the improved or cured rate was 63% in the placebo group versus 87% in the clotrimazole group vs 74% in the econazole group for patients with candida intertrigo and 40%, 81% and 77% respectively for the tinea cruris patients; there was a significant difference in favor of the clotrimazole group versus the placebo patients, but in the clotrimazole groups there was a larger drop-out rate that may have caused bias. The study of Engel [36] compared symmetrical lesions of 25 patients, divided between placebo or amphotericine and found that the results were equally good in both groups; in five days of treatment all lesions were 'clear' or 'almost clear' and no sign of recurrence was found after 14 days, regardless of whether they were treated with placebo or with the therapeutic intervention. Franks [37] included four patients with symmetrical lesions in the large skin folds, that were allocated to either a placebo or candidicine; however, no results were given for the placebo group. Gip [38] studied symmetrical lesions of 18 patients that were treated either with placebo or with dibenzthieen; after 2 weeks 14/18 lesions in the placebo group showed improvement versus 16/18 in the other group. Finally, McMahon [68] allocated symmetrical lesions of 14 patients across five groups; evidently with these sample sizes no differences between groups could be found.

In all, it is remarkable in this group of placebo treatments that many patients showed improvement or healing. Apparently, indifferent treatment may also have some effect; maybe it is caused by attention given to skin folds and or the indifferent cream.

#### Treatment with surgery

We found 15 studies [18,69-82] with surgical treatment. All studies concerned women with macromastia who underwent some kind of reduction mammaplasty. No study compared the surgical intervention to another intervention, but most gave figures about the preoperative and postoperative prevalence of symptoms. It was not always clear if the preoperative prevalences were actually measured preoperatively or in a retrospective way. The mean sample size for these studies was 138 patients (sd 110.4; min-max 33-406; median 90). In all studies there was a clear finding that the percentage of women with inframammary intertrigo decreased substantially after surgery (varying from 80 tot 100%). Despite the fact that these studies did not compare the intervention to another intervention or to a placebo, they do all point in the same direction with considerable effect in rather large populations. So there is at least weak evidence that reduction mammaplasty helps in solving inframammary intertrigo.

#### Other treatments

Nine studies [16,26,60,67,68,83-86] used interventions that could not be categorized in one of the above treatments. In eight of these the intervention was solitary and in one was it a part of a combination preparation. In three [67,68,83] of these eight studies some kind of comparison was made.

The mean sample size of these 8 studies was 17.2 patients (sd 15.3; min-max 3-49; median 11.5).

The products that were studied are melaleuca alternifolia (=tea tree oil) [84], tacrolimus [16], betulin [85], mericleri salt [86], hamamelis virginiana [26], polynoxylin [83], cicalfate [67], water and soap, water and soap and talcum powder or gauze squares or barrier cream or hydrocolloid [68].

The study-authors of the non comparative studies all claimed an effect of their product. In the comparative studies, cicalfate was slightly more effective than eosine [67], while the studies of Alexander [83] and McMahon [68] were too small in scale to find differences.

In all, the studies from this group of treatment were very weakly designed and have too small samples to allow some conclusion about their effectiveness.

## Discussion

This systematic review included no studies about the prevention of intertrigo in the large skin folds and 68 studies about the treatment of this condition. All included studies had a number of serious methodological weaknesses, preventing the drawing of any firm conclusions about the effectiveness of the studied interventions.

The treatments examined mostly concerned the topical application of a pharmaceutical product directed at the treatment of superinfecting micro-organisms. Besides these interventions, 15 studies were found about reduction mammaplasty in women with macromastia, and all pointed in the direction that inframammary intertrigo is reduced by this kind of surgery. So there is at least weak but uniform evidence that reduction mammaplasty helps. However, it is doubtful if inframammary intertrigo on itself constitutes sufficient reason to perform such an invasive intervention.

Every review has the potential to miss relevant studies. For example, it is entirely conceivable that more studies were performed, but were published in journals that are not indexed by any of the databases we checked (e.g. Chinese articles might have been missed). Also we haven't searched databases with conference proceedings or databases with ongoing trials. However, searching 13 databases covering manuscripts in many languages is more than usual for a systematic review.

Furthermore, there is a risk that this review may be flawed by publication bias. Perhaps more studies were performed but that have not been published. This is often the case with studies that have no or only negative effects. If this were to be the case, these studies would certainly not change the mean result of no firm evidence found in this review. Formal testing of publication bias by means of funnel plot analysis was not performed since this test requires a well-defined and well-measured effect size in all studies, which was not the case here.

During this review, we were frequently confronted with unclear descriptions of what study-authors meant by intertrigo, how they diagnosed it and how they measured healing progress. This underlines the conclusion that intertrigo deserves more serious attention from the dermatology field on all aspects from defining to diagnosing, pathophysiology, prevention, treatment and evaluation.

In all, this systematic review clearly demonstrates that intertrigo is a greatly under-researched area in dermatology. This also means that the new guideline for which the review was undertaken, will again have to base its recommendations on expert opinion and not on research evidence.

## Conclusions

There is no evidence at all about the prevention of intertrigo and there is no firm evidence about its treatment. The treatment of intertrigo of the large skin folds has frequently been studied but only in very weakly designed research. Well designed studies are needed. No firm evidence exists as to which recommendations for a clinical guideline can be formulated.

## Competing interests

The authors declare that they have no competing interests.

## Authors' contributions

Both authors contributed equally to the design of the study, inclusion of the studies and data-extraction and synthesis. PM wrote first draft of manuscript, MvH commented on this. Both authors read and approved the final manuscript.

## Pre-publication history

The pre-publication history for this paper can be accessed here:

http://www.biomedcentral.com/1472-6955/9/12/prepub

## Supplementary Material

Additional file 1 **Table 1 Search results**. Click here for file

Additional file 2 **Table 2 Studied interventions**. Click here for file

Additional file 3 **Table 3 Findings antimycotics**. Click here for file

Additional file 4 **Table 4 Findings corticosteroids**. Click here for file

Additional file 5 **Table 5 Findings combinations**. Click here for file
