# Supplementary material for: Prevention and treatment of intertrigo in large skin folds of adults: a systematic review
Source: BMC Nurs. 2010 Jul 13;9:12. doi: 10.1186/1472-6955-9-12 (PMC2918610; doi:10.1186/1472-6955-9-12)
Supplement: Additional file 1 — Table 1 Search results. [file 1472-6955-9-12-S1.DOC]

| **Table 1: Search results** | | |
| --- | --- | --- |
| **DATABASE** | **N**  **hits** | **N unique hits** |
| pubmed | 636 | 198 |
| embase | 732 | 300 |
| cochrane | 24 | 2 |
| cinahl | 22 | 3 |
| Sci | 365 | 44 |
| Picarta | 149 | 56 |
| invert | 12 | 11 |
| NLH | 3 | 3 |
| LILACS | 39 | 31 |
| SCIELO | 1 | 1 |
| IMEMR | 0 | 0 |
| AMED | 5 | 1 |
| CAMbase | 1 | 0 |
| **TOTAL** | **1989** | **1124** |
