# Supplementary material for: Prevention and treatment of intertrigo in large skin folds of adults: a systematic review
Source: BMC Nurs. 2010 Jul 13;9:12. doi: 10.1186/1472-6955-9-12 (PMC2918610; doi:10.1186/1472-6955-9-12)
Supplement: Additional file 2 — Table 2 Studied interventions. [file 1472-6955-9-12-S2.DOC]

| **Table 2: Studied interventions** | | |
| --- | --- | --- |
| **Type of intervention** | **Number of studies**  **(in which treatment category was applied either as a sole treatment or as a component of a combination** | **Number of studies**  **(in which treatment category was applied as a sole treatment)** |
| *Antimycotics* | 38 | 25 |
| *Corticosteroids* | 17 | 4 |
| *Antibiotics* | 8 | 0 |
| *Antiseptics* | 5 | 1 |
| *Combinations* | 18 | 18 |
| *Placebo* | 6 | 6 |
| *Surgery* | 15 | 15 |
| *Other*  *(e.g. tea tree oil, hamamelis, ….)* | 9 | 8 |
