# Supplementary material for: Prevention and treatment of intertrigo in large skin folds of adults: a systematic review
Source: BMC Nurs. 2010 Jul 13;9:12. doi: 10.1186/1472-6955-9-12 (PMC2918610; doi:10.1186/1472-6955-9-12)
Supplement: Additional file 3 — Table 3 Findings antimycotics. [file 1472-6955-9-12-S3.DOC]

| **Table 3: Findings in included studies on antimycotics** | | | | | |
| --- | --- | --- | --- | --- | --- |
| **Type antimycotic** | **Study design** | | | | **Effects** |
|  | **Non- comparative** | **Comparison with same product (other dose/frequency)** | **Comparison with other product** | **Comparison with**  **placebo** |  |
| ***antimycotic antibiotics*** |  |  |  |  |  |
| amphotericine |  |  |  | (Engel 1965)^36^  (n=25) | Symmetrical lesions of 25 patients were allocated to amphotericine or placebo. All lesions from both groups healed within 5 days |
| nystatine | (Grupper 1954)^40^  (n=12) |  | VERSUS fluorocytosine (Gisslen 1974)^39^  (n=24) |  | - in the Grupper study all 12 patients healed within 4 days application of nystatine.  - in the Gisslen study symmetrical lesions of 24 patients were allocated; 19/24 lesions treated with fluorocytosine showed considerable improvement or healed within 2 weeks versus 21/24 treated with nystatine |
| candidicin |  | Candidicin in water VERSUS  candidicin in glycol  (Franks 1954)^37^  (n=4) |  | (Franks 1954)^37^  (n=4) | No conclusions possible due to the small N (n=4) and no results presented of the placebo group |
| pimaricin |  |  | VERSUS  pimafucort  (Alteras 1969)^30^  (n=22) |  | After 3 to 8 weeks treatment with pimaricine 14/22 patients healed and the other 8 showed improvement; no results presented about the treatment with pimafucort |
| ***imidazolen*** |  |  |  |  |  |
| ketoconazole | ketoconazole per os (Puiatti 1986^28^ (n=9); Grigoriu 1982^27^ (n=5))  ketoconazole local (Amerighi 1984)^31^  (n=22) |  |  |  | - In the Puiatti study and the Grigoriu study all 9, c.q. all 5 patients healed after treatment with ketoconazole orally during 6-8 weeks  - in Amerighi study 18/22 patients healed within 2 to 6 weeks of treatment with ketoconazole powder; the other 4 showed improvement and in all patients symptom burden decreased |
| micozanole | (Varma2007)^57^  (n=10) |  |  |  | All 10 patients improved within 12 weeks of treatment with miconazole gel |
| bifonazole | (Vogt1985)^58^  (n=52) |  |  |  | 96% of the patients showed very good results, both based on clinical and microscopically assessment after 3 weeks of treatment with bifonazole |
| clotrimazole |  |  | VERSUS  econazole (Cullen1984^20^; n=38) (Miura1979^22^; n=245) | (Cullen1984^20^; n=38) (Miura1979^22^; n=245) | - in the Cullen study 10/14 patients from the econazole group healed versus 11/18 from the clotrimazole group versus 1/6 from the placebo group  - in the Miura study 74% in the subgroup of candida-intertrigo patients healed after treatment with econazole versus 87% after treatment with clotrimazole versus 63% after placebo; in the subgroup of tinea cruris patients the healing rate was 77% after econazole treatment versus 81% after clotrimazole and 40% in the placebo group |
| econazole | (Hempel1975)^43^  (n=13)  (Siboulet1976)^52^  (n=9) | Econazole milk  VERSUS  econazole cream (Scherwitz1977^49^; n=34)  econazole spray VERSUS  econazole lotion VERSUS  econazole milk (Schwarz1975^51^; n=205) | VERSUS  clotrimazole (Cullen1984^20^; n=38) (Miura1979^22^; n=245) | (Cullen1984^20^; n=38) (Miura1979^22^; n=245) | - in the Hempel study 11/13 patients healed after 7-28 days treatment with econazole  - in the Siboulet study 8/9 patients showed good to excellent respons on econazole after a mean of 8 days of treatment  - in the studies of Scherwitz and Schwarz no differences were found between the different application forms of econazole; in all a cure-rate of approximately 90% was reached  - in the Cullen study 10/14 patients healed from the econazole group versus 11/18 from the clotrimazole group versus 1/6 from the placebo group  - in the Miura study 74% in the subgroup of candida-intertrigo patients healed after treatment with econazole versus 87% after treatment with clotrimazol versus 63% after placebo; in the subgroup of tinea cruris patients the healing rate was 77% after econazole treatment versus 81% after clotrimazole and 40% in the placebo group |
| thiabendazole | (Thomas1971)^56^  (n=1) |  |  |  | No conclusions possible due to the small N (n=1) |
| tioconazole | (Somorin1985; n=4)^53^  (Taube1995; n=18)^55^ |  |  |  | In the Somorin all four and in the Taube study 16/18 patients healed |
| ***triazolen*** |  |  |  |  |  |
| fluconazole |  | Fluconazole per os 150mg/week  VERSUS  fluconazole per os 50mg/day (Nozickova1998)^23^  (n=52) |  |  | There was no difference between the weekly and the daily dose schema; in both cases 80-90% healed within 1 month |
| ***allylamine*** |  |  |  |  |  |
| naftifin | (Otcenasek1993)^45^  (n=5) |  |  |  | No conclusions possible due to the small N (n=5) |
| ***overige antimycotica*** |  |  |  |  |  |
| cyclopyroxolamine | (Radovic1990; n=13)^46^ (Szepes1986; n=9)^54^ |  |  |  | In both studies with cyclopyroxolamine all patients healed within 1 month |
| fluorocytosin |  |  | VERSUS  nystatine (Gisslen1974)^39^  (n=23) |  | In the Gisslen study symmetrical lesions of 24 patients were allocated; 19/24 lesions treated with fluorocytosine showed considerable improvement or healed within 2 weeks versus 21/24 treated with nystatine |
| dibenzthieen |  |  |  | (Gip1966)^38^  (n=18) | Symmetrical lesions of 18 patients were allocated; 16/18 from the dibenzthieen-group improved versus 14/18 form the placebo group |
| buclosamide | (Guha1974)^41^  (n=20) |  |  |  | 18/20 patients showed good response to buclosamide within 3 weeks |
