# Supplementary material for: Prevention and treatment of intertrigo in large skin folds of adults: a systematic review
Source: BMC Nurs. 2010 Jul 13;9:12. doi: 10.1186/1472-6955-9-12 (PMC2918610; doi:10.1186/1472-6955-9-12)
Supplement: Additional file 4 — Table 4 Findings corticosteroids. [file 1472-6955-9-12-S4.DOC]

| **Table 4: Findings in included studies on corticosteroids** | | | | | |
| --- | --- | --- | --- | --- | --- |
| **Type of corticosteroid** | **Study design** | | | | **Effects** |
|  | **Non- comparative** | **Comparison to same product (other dose/frequency)** | **Comparison to other product** | **Comparison to**  **placebo** |  |
| betamethasone valerate 0.01% | (Becker1973)^61^ (n=8) |  |  |  | 6/8 patients had good response |
| diflucortolne valerate 0.1% | (Bergson1977)^25^ (n=7) |  |  |  | 6/7 patients had good response within 6 weeks |
| triamcinolone crème | (Marton1977)^64^ (n=18) |  |  |  | All patients healed |
| hydrocortisone crème 1% |  |  | Versus  hydrocortisone  + miconazole  (Hedley1990)^21^  (n=78) |  | Symptoms and symptom burden decreased or disappeared in all patients from both groups |
