# Supplementary material for: Prevention and treatment of intertrigo in large skin folds of adults: a systematic review
Source: BMC Nurs. 2010 Jul 13;9:12. doi: 10.1186/1472-6955-9-12 (PMC2918610; doi:10.1186/1472-6955-9-12)
Supplement: Additional file 5 — Table 5 Findings combinations. [file 1472-6955-9-12-S5.DOC]

| **Table 5: Findings in included studies with a combination intervention** | | | | | |
| --- | --- | --- | --- | --- | --- |
| **Type of combination intervention** | **Design** | | | | **Effects** |
|  | **Non- comparative** | **Comparison with same product (other dose/frequency)** | **Comparison with other product** | **Comparison with**  **placebo** |  |
| Exosterol  = phenylhydrargyri boras + dexamethason | Hofer1974^63^  (n=25) |  |  |  | 16/25 patients healed within 1 month and the other improved |
| Fluocinolon Acetonide + neomycine | Gatti1963^62^  (n=2) |  |  |  | Both intertrigo- patients improved after 1 week of treatment |
| Stapolidex  =staphylomycine + polymixine + dexamethason | Vallette1968^65^  (n=2) |  |  |  | Both intertrigo- patients improved a bit after an unspecified period |
| Pevaryl  =econazole + zinkoxide paste | Wurster1993^60^  (n=124) |  |  |  | After 2 weeks of treatment 90% of patients achieved improvement or cure |
| Pevisone  =econazole + triamcinolon crème | Baran1979^34^  (n=73)  Masse1980^44^  (n=8) |  |  |  | Approximately 90% healed or had a strong improvement within 1 month |
| Daktacort  =miconazole + hydrocort | Guilhou1979^42^  (n=3) |  | Versus hydrocortisone  Hedley1990^21^  (n=78) |  | Symptom(burden) decreased strongly in all patients from both groups in the Hedley-study |
| Logamel  = triclosan + Flumethasone pivalate | Reiffers1981^47^  (n=15) |  |  |  | 13/15 patients healed within 2 weeks to 2 months |
| Kenacomb  =Triamcinolon + neomycine + gramicidine + nystatine | Rosanove1967^48^  (n=37) |  |  |  | All patients healed within 4 weeks, of which half within 1 week |
| Flupredniliden + gentamycine + hydroxiquinolin | Anonymous1989^32^  (n=15) |  |  |  | 87% healed within 2 weeks |
| Pimafucort  =neomycine + bacitracine + natamycine + dexamethason | Durand1975^35^  (n=4) |  | Versus  Pimaricine  Alteras1969^30^  (n=22) |  | - in the Durand study all patients healed;  - the study of Alteras did not present results of the treatment with pimafucort in the intertrigo-patients |
| Topsym  =fluocinonide + neomycin + gramicidin + nystatine ointment | Schmidt1975^50^  (n=29) |  |  |  | 27/29 patients had good results within 3 weeks |
| Timodine  =nystatine + benzalkonium + hydrocort + diemthicon | Almeyda1974^29^  (n=27) |  |  |  | 78% had good to excellent result |
| Trixarol  =bufexamac + nystatine + neomycine | Aussems1972^33^  (n=8) |  |  |  | 7/8 patients healed within 4 weeks and the other improved a lot |
| Fluocortolon + clorchinaldo |  |  | VERSUS  Betamethasone + clorossina  Venier1982^66^  (n=12) |  | All patients showed good response to both treatments |
| Betamethasone + clorossina |  |  | VERSUS  Fluocortolon + clorchinaldo  Venier1982^66^  (n=12) |  | All patients showed good response to both treatments |
| AI307  =dichloroxychinaldin + jodchlroxychinolin |  | Wanic1967^59^  (n=6) |  |  | There was some effect in both the powder and the ointment form, but no conclusions possible due to the small N |
